# Automated assessment of left ventricular function and mass using heart deformation analysis: initial experience in 160 older adults

**DOI:** 10.1186/1532-429X-18-S1-P38

**Published:** 2016-01-27

**Authors:** Kai Lin, Jeremy Collins, Michael Markl, James C Carr

**Affiliations:** grid.465264.7Radiology, Northwestern University, Chicago, IL USA

## Background

Cardiac aging, which can result in subclinical alterations in the heart, is considered a condition that bridges elderly and the incidence of cardiovascular events. Left ventricular (LV) ejection fraction (LVEF) and mass (LVM) are indices presenting ventricular dysfunction and hypertrophy, two important indicators in cardiovascular risk estimation. Heart deformation analysis (HDA) is a recently developed imaging processing technique for the description of global and regional myocardial function and motion on cine MRI. Using a deformation image registration (DIR) algorithm, HDA is able to automatically track the shape of LV through the entire cardiac cycle by calculating deformation fields (forward and backward) of myocardium tissue among sequential cardiac time frames. As such, global cardiac indices (LVEF, CO and LVM) as well as regional myocardial motion indices (displacement, velocity, strain and strain rate) can be extracted from cine images using a semi-automatic "one-stop-shop" analysis with minimal user interaction. However, to date the capability of HDA tool for the evaluation of LV global function and morphology has not been validated in a larger clinical study cohort. Therefore, the aim of the present study was to assess the accuracy of automated quantification of left ventricular function and mass based on HDA in asymptomatic older adults by using a standard LV global function analysis as the reference standard.

## Methods

This study complied with HIPAA regulations. Following the approval of the institutional review board (IRB), 160 asymptomatic older participants were recruited for cardiac MRI including two-dimensional (2D) cine images covering the entire left ventricle (LV) in short-axis view. Data analysis included the calculation of left ventricular ejection fraction (LVEF), mass (LVM) and cardiac output (CO) using HDA and standard global cardiac function analysis (delineation of end systolic and diastolic LV epi- and endo-cardial borders). The agreement between methods was evaluated using intra-class correlation coefficient (ICC) and coefficient of variation (CoV).

## Results

There was good agreement for LVEF (ICC = 0.552, CoV = 10.5%), CO (ICC = 0.773, CoV = 13.5%) and LVM (ICC = 0.859, CoV = 14.5%) acquired with standard method and HDA. There was a systemic bias towards lower LVEF (62.8% ± 8.3% vs.69.3% ± 6.7%, p < 0.001) and CO (4.4 ± 1.0 L/minute vs. 4.8 ± 1.3 L/minute, p < 0.001) by HDA compared to the standard technique. Conversely, HDA overestimated LVM (114.8 ± 30.1 g vs. 100.2 ± 29.0g, p < 0.001) as compared to the reference method.

## Conclusions

HDA has the potential to measure LVEF, CO, and LVM without the need for user interaction based on standard cardiac 2D Cine images.

**Figure 1:**
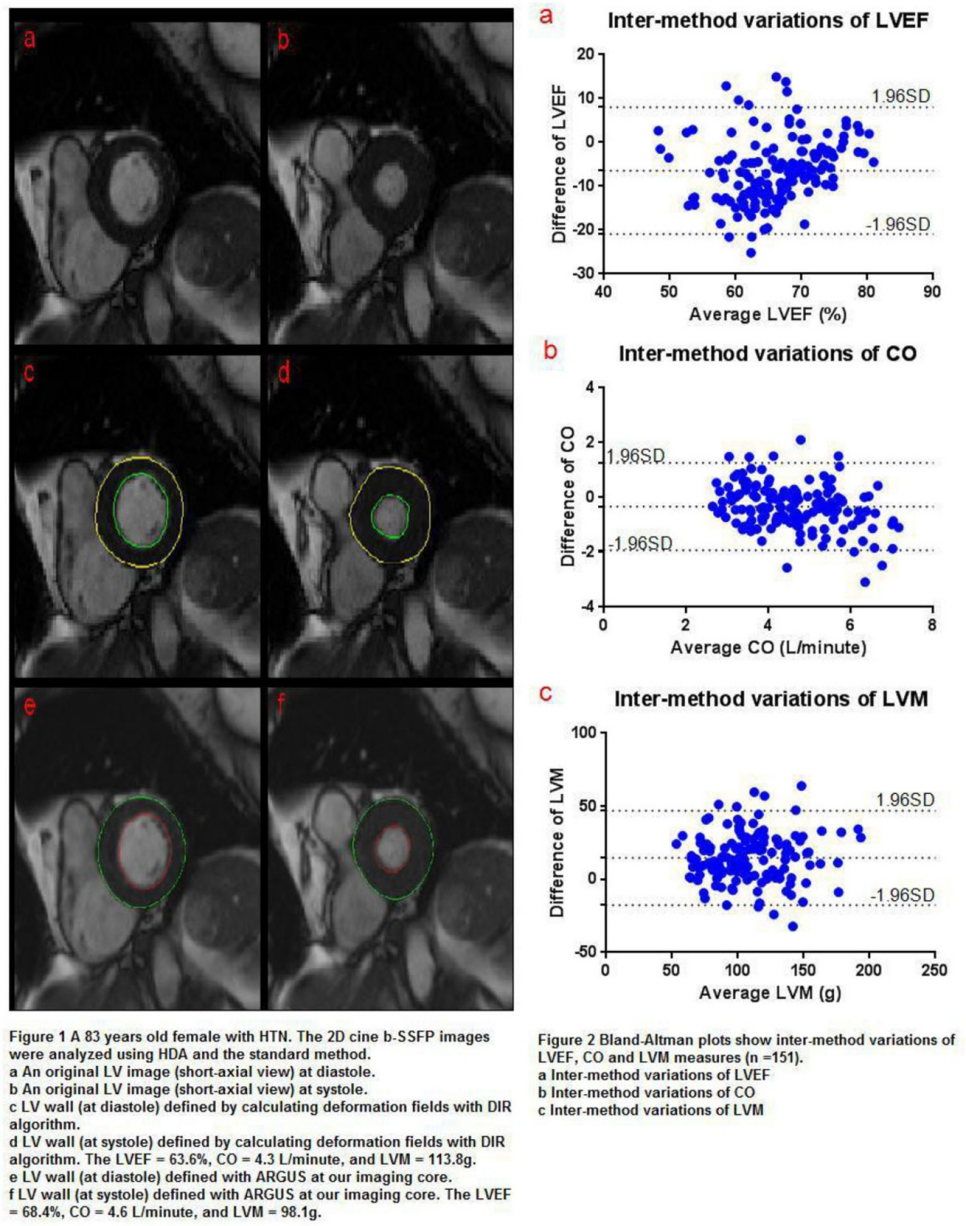
Figure 1

